# GC-MS Metabolomics Reveals Distinct Profiles of Low- and High-Grade Bladder Cancer Cultured Cells

**DOI:** 10.3390/metabo9010018

**Published:** 2019-01-18

**Authors:** Daniela Rodrigues, Joana Pinto, Ana Margarida Araújo, Carmen Jerónimo, Rui Henrique, Maria de Lourdes Bastos, Paula Guedes de Pinho, Márcia Carvalho

**Affiliations:** 1 UCIBIO/REQUIMTE, Department of Biological Sciences, Laboratory of Toxicology, Faculty of Pharmacy, University of Porto, 4050-313 Porto, Portugal; jipinto@ff.up.pt (J.P.); ana.margarida.c.araujo@gmail.com (A.M.A.); mlbastos@ff.up.pt (M.d.L.B.); pguedes@ff.up.pt (P.G.d.P.); 2 Cancer Biology & Epigenetics Group, Research Center (CI-IPOP) Portuguese Oncology Institute of Porto (IPO Porto), 4200-072 Porto, Portugal; carmenjeronimo@ipoporto.min-saude.pt (C.J.); rmhenrique@icbas.up.pt (R.H.); 3 Department of Pathology and Molecular Immunology-Biomedical Sciences Institute (ICBAS), University of Porto, 4050-313 Porto, Portugal; 4 Department of Pathology, Portuguese Oncology Institute of Porto (IPO Porto), 4200-072 Porto, Portugal; 5 UFP Energy, Environment and Health Research Unit (FP-ENAS), University Fernando Pessoa, 4249-004 Porto, Portugal

**Keywords:** bladder cancer, cancer progression, in vitro, metabolomic signatures, endometabolome, GC-MS, metabolic pathways

## Abstract

Previous studies have shown that metabolomics can be a useful tool to better understand the mechanisms of carcinogenesis; however, alterations in biochemical pathways that lead to bladder cancer (BC) development have hitherto not been fully investigated. In this study, gas chromatography-mass spectrometry (GC-MS)-based metabolomics was applied to unveil the metabolic alterations between low-grade and high-grade BC cultured cell lines. Multivariable analysis revealed a panel of metabolites responsible for the separation between the two tumorigenic cell lines. Significantly lower levels of fatty acids, including myristic, palmitic, and palmitoleic acids, were found in high-grade versus low-grade BC cells. Furthermore, significantly altered levels of some amino acids were observed between low- and high-grade BC, namely glycine, leucine, methionine, valine, and aspartic acid. This study successfully demonstrated the potential of metabolomic analysis to discriminate BC cells according to tumor aggressiveness. Moreover, these findings suggest that bladder tumorigenic cell lines of different grades disclose distinct metabolic profiles, mainly affecting fatty acid biosynthesis and amino acid metabolism to compensate for higher energetic needs.

## 1. Introduction

Bladder cancer (BC) is the second most common genitourinary malignancy and one of the deadliest cancers worldwide [1]. BC can be classified as low-grade (LG) or high-grade (HG) according to histopathological characteristics [2,3], with low-grade meaning that the differentiation of the tumor is more similar to normal than high-grade. Importantly, HG BC is more aggressive and prone to invasion than LG [2]. Although significant progresses in unveiling new diagnostic strategies based on molecular markers have been made [4,5], their high cost and flaw in detecting early BC do not offer advantage over classical ones [6], thus hindering their clinical application [5]. Therefore, there is an urgent need for discovering early, specific, cost-effective, and non-invasive diagnosis methods, so that therapeutics can be more effective.

Metabolomics has proven to be a promising and alternative tool for early cancer detection, through the comprehensive analysis of alterations in metabolite levels that can be translated into biomarkers. These metabolite signatures reflect the biological activity of each cancer cell type, which brings the possibility of distinguishing unique dysregulations in metabolic pathways characteristic of different cancer subtypes [7]. This approach has been already applied to several cancers including those of breast [8], ovary [9], kidney [10], colorectum [11], and hepatocellular carcinomas [12], through the screening and detection of metabolite levels in human urine, feces, or biofluids, which represents an advantage over classical invasive diagnosis methods [13]. Nevertheless, discrepancies among studies that aimed to investigate cancer biomarkers for early detection through metabolomic analysis are compelling, hampering the development of a diagnostic tool based on such molecules. This particularly applies to the case of BC, for which studies based on metabolomic diagnosis biomarkers are controversial and limited [3]. For these reasons, translatability to clinical settings has been hindered. Therefore, more studies focusing on early detection and the progression of BC are urgently needed. 

Recently, studies on metabolomics have also focused on in vitro approaches to obtain further information about metabolic pathways that lead to BC progression [3]. In vitro cell culture systems represent the least complex disease model and have a number of advantages over tissue or biofluid analysis, including simpler and controllable experimental settings, less variability among samples, reduction in animal testing, and the provision of better insight into metabolic changes [14]. Nevertheless, this study model has only comprised immortalized cell lines and primary cell cultures so far, which leads to some limitations mainly related to extrapolation to in vivo systems [15], the inability to mimic the environment and communication between surrounding cells [16], the need of routine evaluation, and a careful interpretation of results, since metabolic perturbations can be caused by changes in the culture cell medium rather than to the disease itself [14].

The application of metabolomic approaches, using different disease models, has led to the discovery of several metabolites whose levels are altered in BC cells (see review by Rodrigues et al. [3]) which are involved in important biochemical pathways that produce energy, including glycolysis, tricarboxylic acid (TCA) cycle, fatty acid β-oxidation, and amino acid metabolism. Nevertheless, there is a clear gap in the search for metabolites that can be used to distinguish different grades of BC, particularly in in vitro studies, since past research has mostly focused on distinguishing normal and benign cancer samples. There are very few studies conducted using a metabolomics approach to distinguish LG from HG BC, all of which applied to either human plasma/serum or urine [17,18,19]. However, to the best of our knowledge, no study has yet investigated the endometabolome signatures of BC cultured cell lines with different tumor grades, making our in vitro study a pivotal one in searching for metabolic differences between LG and HG transitional cell carcinoma (TCC) of the bladder. In this study, we applied gas chromatography-mass spectrometry (GC-MS)-based metabolomics to determine the endometabolome signatures of two BC cell lines of different tumor grades. This approach not only allowed for a demonstration of the potential of metabolomics analysis to discriminate BC cells according to tumor aggressiveness but also extended the knowledge on the metabolic alterations between LG and HG bladder TCC, an evaluation which is lacking among published in vitro studies.

## 2. Material and Methods

### 2.1. Chemicals

Eagle’s minimum essential medium (MEM) supplemented with l-glutamine, *N*-trimethylsilyl-*N*-methyl trifluoroacetamide (MSTFA, ≥98.5%), desmosterol, and l-norvaline was purchased from Sigma-Aldrich (St. Louis, MO, USA). Penicillin, streptomycin, trypsin, and fetal bovine serum (FBS) were purchased from Invitrogen (Karlsruhe, Germany). Phosphate buffer solution (PBS) was purchased from Biochrom (Merck, Berlin, Germany); methanol (99.9%) and dichloromethane (99%) were purchased from VWR (Leuven, Belgium). All chemicals were of analytical grade and were dissolved in ultrapure water unless otherwise indicated.

### 2.2. Cell Lines and Culture Conditions

The BC cell lines 5637 and J82 were obtained from American Type Culture Collection (ATCC; Manassas, VA, USA). Both cell lines were derived from transitional cell carcinoma of the human urinary bladder, with 5637 being classified as grade II and J82 as grade III (stage pT3) [20]. BC cell lines were cultured in MEM to ensure optimal cell growth and maintenance of epithelial cell characteristics. The culture medium was prepared as indicated by the manufacturer and supplemented with 10% FBS and 100 units·mL^−1^ penicillin/100 μg·mL^−1^ streptomycin. All cell lines were routinely tested for *Mycoplasma* spp. contamination (PCR Mycoplasma Detection Set, Clontech Laboratories).

### 2.3. Sample Collection 

The experiments were carried out during five passages, with triplicates for each passage, after an adaptation stage of at least three passages for both cell lines. Cells were grown for 48 h in T75 culture flasks to near confluence. The culture medium was discarded and cells were gently washed with 2 mL PBS to remove all medium. Cold methanol (3 mL) was then added to each flask to effectively quench the metabolism of cells. Subsequently, cells were scraped off the flasks on ice, transferred to a falcon tube and centrifuged for 10 min at 3000 *g* at 4 °C. The supernatant was collected and stored at −80 °C until analysis. 

### 2.4. Sample Preparation for GC-MS Analysis

Each sample (1 mL) was transferred into a glass vial, followed by the addition of 10 μL desmosterol (1 mg/mL) and norvaline (1 mg/mL) as internal standards. These compounds, which were added in equal amounts to all samples, were used to monitor the performance of the of GC-MS acquisition (injection issues and retention time deviation). Subsequently, the mixture was vortex-mixed at high speed for 1 minute and carefully evaporated to dryness at room temperature under a gentle stream of nitrogen gas. The derivatization process was adapted from a previous work performed in our laboratory [21,22], which was carried out by adding 50 μL MSTFA and 50 μL dichloromethane to the dried extract, followed by vortex-mixing at high speed for 1 minute and incubation for 30 min at 80 °C. Then, the derivatized solution was cooled and transferred into screw-top autosampler vials for subsequent GC-MS analysis. In addition, quality control samples (QCs) were prepared as a pool of all samples in the study and divided into aliquots to avoid the repeated freeze–thaw cycles. QCs were derivatized using the same protocol applied for samples. 

### 2.5. GC-MS Analysis: Equipment and Conditions

The endometabolome profiles of J82 and 5637 cells were obtained using an EVOQ 436 GC system (Bruker Daltonics, Fremont, CA) coupled to a SCION TQ mass detector, a Bruker Daltonics MS workstation software (version 8.2), and a Combi-PAL autosampler (Varian Pal Autosampler, Switzerland), as described previously [22]. Briefly, random injection of all samples was performed and a QC sample was also injected in every four samples, under the same conditions, for a total of seven QCs. Chromatographic separation was obtained by using a GC fused silica capillary column BR-5ms (5% phenyl, 95% dimethyl polysiloxane, 30 m × 0.25 mm × 0.25 μm) (Bruker Daltonics, Freemont, CA, USA). The injector temperature was 250 °C and samples (1 μL) were introduced in split mode with a 1:5 ratio. Helium C-60 (Gasin, Portugal) was the carrier gas with a flow rate of 1.0 mL/min. The program set for the column temperature was as follows: initial temperature at 70 °C held for 2 min, then ramped at 15 °C/min to 250 °C, held for 2 min, and finally increased at 10 °C/min to 300 °C, then held for 8 min, having a total duration of 29 min per run. The MS detector was functioning in Electron Impact (EI) mode. The transfer line temperature was 230 °C, the manifold temperature was 40 °C, and the ion source temperature was 250 °C. The mass range selected was 50–600 *m*/*z*, with a scan rate of 6 scans per second. The resolution and intensity of the chromatographic peaks among all samples were monitored using norvaline and desmosterol as internal standards. 

### 2.6. GC-MS Data Pre-Processing

All raw data files obtained from GC-TQ-MS were exported as Computable Document Format (CDF) files and pre-processed to convert instrumental data sets into a manageable format for data analysis and remove any biases such as background, noise, and retention time (RT) fluctuations over a set of samples. Data pre-processing was performed using the software MZmine 2.21 [23]. The parameters used in these steps were set as follows: RT range 2.8–29.0 min; *m*/*z* range 50–600; MS data noise level 2.5 × 10^4^; *m*/*z* tolerance 0.5; chromatogram baseline level 2.0 × 10^4^; peak duration range 0.05–0.50 min. Chromatographic peaks regarded as trash or irrelevant were manually removed from the data matrix, as well as all peaks with a relative standard deviation (RSD) ≥30% across all QCs. Normalization of the data was performed by determining the mean of the chromatogram’s area of each set of triplicates, which was subsequently divided by the total area. Ultimately, statistical analysis was performed with the resulting normalized peak areas. 

### 2.7. Metabolite Identification

The identification of metabolites in GC-TQ-MS chromatograms was performed by comparing the retention indices (RI) and mass spectra fragmentation patterns of each compound with the RI and mass spectra present in the National Institute of Standards and Technology (NIST) spectral library version 14 (Gaithersburg, MA, USA). The reverse match obtained by NIST 14 was also considered and the identification was obtained when a value of 700 or above was achieved. Compounds for which no satisfactory match was found were listed as “unknown i” (*i* = 1,2,3…) according to their RT in ascending order. When possible, the compound identification was confirmed through comparison of their RTs and mass spectra with that obtained from pure standards. Pathway and metabolite information were extracted from the Human Metabolome Database (HMDB) [24] and the Kyoto Encyclopedia of Genes and Genomes (KEGG) [25].

### 2.8. Metabolic Pathway Analysis

To explore which metabolic pathways were altered and contributed the most to the separation between HG J82 and LG 5637 cell lines, Metabolite Set Enrichment Analysis (MSEA) [26], specifically the pathway over-representation analysis, was performed using the freely available online software Metaboanalyst 4.0 [27], where biologically meaningful patterns were investigated using the set of significantly different metabolites (compound names).

### 2.9. Statistical Analysis

Principal component analysis (PCA) and partial least squares discriminant analysis (PLS-DA) were applied to the final matrix scaled to pareto (Par) [28], using SIMCA-P 13.0.3 (Umetrics, Umea, Sweden). The R^2^X (variance explained by the *X* matrix), R^2^Y (variance explained by the *Y* matrix), and Q^2^ (goodness of prediction or prediction power) parameters, obtained by 7-fold cross validation, were used to evaluate the model robustness (SIMCA-P 13.0.3). The PLS-DA loadings plot and the variable importance to the projection (VIP > 1) of each variable were used to assess the variables (*m*/*z*-RT pairs) responsible for group separation. For subsequent analyses, the peak area of all detected derivatives from the same metabolite were summed, as recommended in the literature [29].

The statistical significance of relevant compounds identified in the PLS-DA loadings plots was assessed using the unpaired Mann–Whitney test (non-normally distributed data), in GraphPad Prism version 6 (GraphPad Software, San Diego, CA, USA). This software was also used to determine the area under the curve (AUC) for each metabolite. For each model, the discriminative compounds were considered statistically significant when *p* < 0.05 (confidence level 95%). Effect size [30] was computed for compounds with *p* < 0.05 and corrected for small sample sizes. 

## 3. Results and Discussion

In this study, the endometabolome profiles of the LG BC cell line 5637 and the HG BC cell line J82 were analyzed to search for metabolites with the potential to assess tumor aggressiveness. Representative GC-MS chromatograms of the in vitro tumorigenic cells are shown in Supplementary Figure S1. A total of 37 metabolites were identified across all samples, except for one metabolite which remained unknown (Supplementary Table S1). Most of the identified metabolites belong to the classes of amino acids, fatty acids, and organic acids or derivatives. Less represented classes included amino alcohols, monosaccharides, sterols, and sugar alcohols. The general characteristics of the metabolites detected in the endometabolome of BC cell lines, such as RT, RI, characteristic ions (*m*/*z*), metabolite identification method, HMDB identification, and the main pathways in which they are involved, are summarized in Supplementary Table S1. Whenever available, BC studies in which those metabolites have been previously found are also described. 

**Figure 1:**
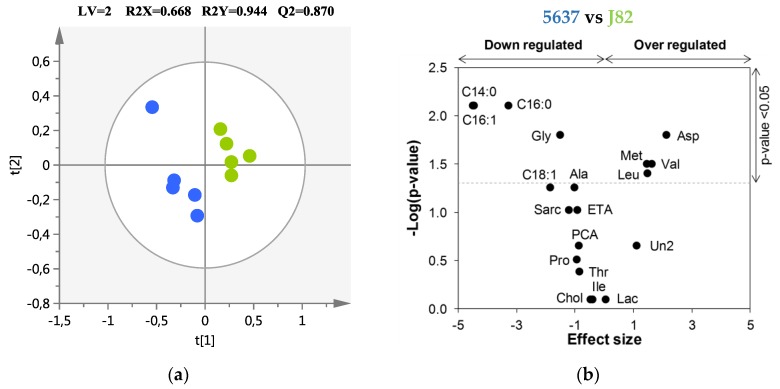
(**a**) Partial least squares discriminant analysis (PLS-DA) scores scatter plot obtained for the cancer cell lines 5637 (*n* = 5, low-grade, (●) and J82 (*n* = 5, high-grade, (●). The ellipses indicate the 95% confidence limit of the model; (**b**) Volcano plot showing the metabolites contributing to the discrimination between high-grade (HG) J82 and low-grade (LG) 5637 cells. Horizontal dashed line indicates the significance level (*p* < 0.05). Three-letter codes are used for amino acids. C14:0, myristic acid; C16:0, palmitic acid; C16:1, palmitoleic acid; Chol, cholesterol; ETA, ethanolamine; Lac, lactic acid; PCA, pyroglutamic acid; Sarc, sarcosine; Un2, unknown 2.

PCA was first performed to explore the metabolic differences between LG BC and HG BC cell endometabolomes, showing a tendency to separate both (R^2^X = 0.690, Supplementary Figure S2). PLS-DA confirmed a clear separation between LG BC and HG BC (Figure 1a) with a good prediction power (Q^2^ = 0.870). Eight metabolites were identified as important for discriminating between LG BC and HG BC cell lines, as represented in the Volcano plot (Table 1, Figure 1b). Glycine, myristic, palmitic, and palmitoleic acids were found to be significantly decreased in HG J82 cells, whereas leucine, methionine, valine, and aspartic acid were found to be significantly increased in the HG compared to the LG cell line. 

**Figure 2:**
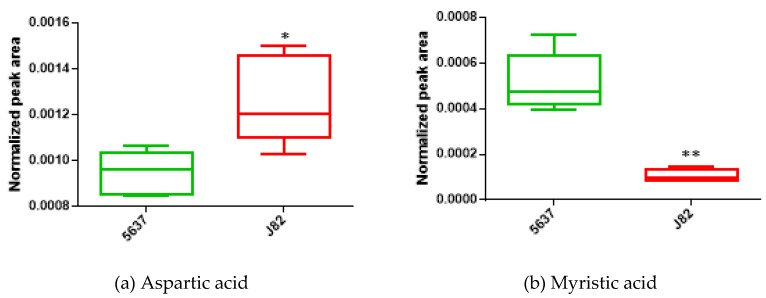
Boxplots of (**a**) aspartic acid and (**b**) myristic acid, two metabolites found to be significantly altered between the LG bladder cancer cell line 5637 (*n* = 5) and the HG bladder cancer cell line J82 (*n* = 5). * *p* < 0.05; ** *p* < 0.01.

**Figure 3:**
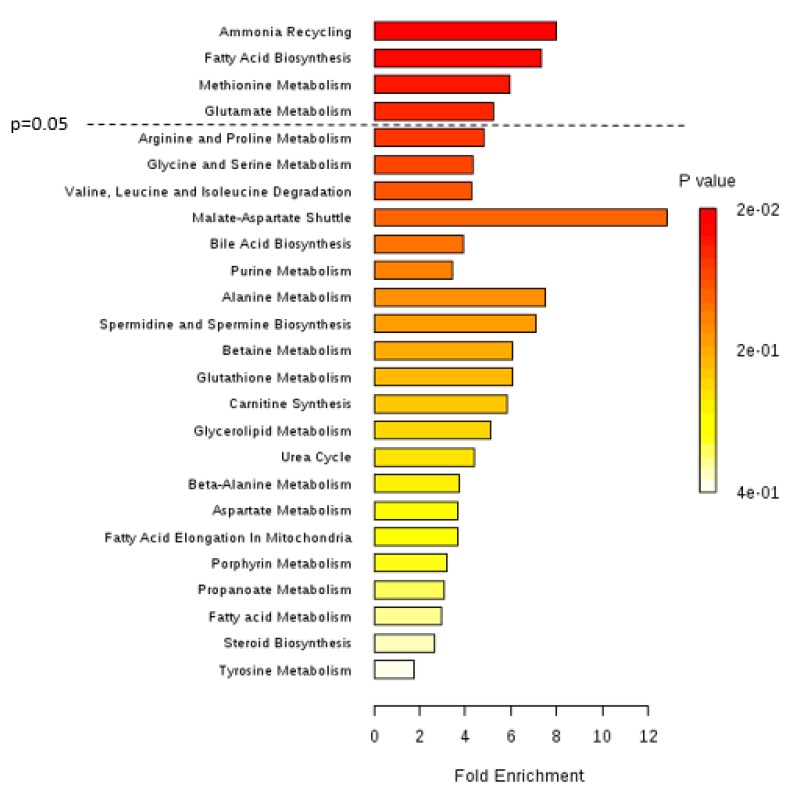
Summary plot for metabolite set enrichment analysis (MSEA) performed for the HG bladder cancer (BC) cell line (J82) versus the LG BC cell line (5637), where metabolite sets are ranked according to the Holm *p*-value. The horizontal bar graph summarizes metabolic pathways that were different between HG and LG bladder cancer cells. Significantly different pathways include ammonia recycling, fatty acid biosynthesis, and methionine and glutamate metabolisms.

Boxplots of two of the most significantly altered metabolites between the two cancer cell lines are represented in Figure 2a (aspartic acid) and Figure 2b (myristic acid). Figure 3 shows the metabolic pathways that are significantly disturbed between the two BC cell lines, which will be discussed in detail below.

The levels of myristic, palmitic, and palmitoleic acids were significantly decreased in the higher tumor grade cell line, which is concordant with the well-known fact that cancer cells, as they grow into more advanced stages, change their energetic requirements to keep on growing and proliferating. This is because several important biological processes, such as the synthesis of DNA and proteins or the production of new cellular components, need to be maintained and enhanced as the tumor becomes more aggressive [31,32]. Fatty acids (FAs), when metabolized by cells, yield great amounts of energy that serve as fuel for several cellular processes such as the TCA cycle and β-oxidation [33]. Moreover, FAs are also involved in other cellular processes, particularly cell signaling and cell membrane integrity. To the best of our knowledge, no previous studies have reported alterations in the levels of these three FAs according to BC grading. Therefore, our results offer an important insight into the metabolic differences between tumor grades and clearly demonstrate that LG and HG BC cells generate a different in vitro signature that reflects the distinct metabolic needs of those cancer cells, with advanced grades requiring more FA consumption for survival and continuous growth and proliferation [3].

Referring to amino acids, glycine was found to be decreased in HG BC cells (J82) compared to LG BC cells (5637). Corroborating our findings, previous studies by Dettmer et al. [34] and Cao et al. [35] observed lower levels of glycine in the serum of patients with advanced BC compared to patients with LG BC. Glycine may play a critical role in tumor progression to higher grades [36], being involved not only in amino acid metabolism but also in purine and glutathione metabolism [3]. This suggests that these two pathways might be augmented in HG cancer cells and glycine may be highly used in order to either produce more DNA/RNA components, proteins, or antioxidant metabolites. Glycine is also involved in ammonia recycling (Figure 3), being used to give rise to ammonia through the action of the glycine cleavage system [37]. Upregulation of this ammonia recycling-related pathway might explain the decrease in glycine levels as BC progresses, but further studies are required to confirm this hypothesis.

Apart from glycine, four other amino acids were significantly altered between the two BC cell lines, namely aspartic acid, leucine, valine, and methionine, which may indicate alterations in amino acid metabolism (Figure 3) and consequently in the pathways in which these amino acids are involved, namely protein biosynthesis and the TCA cycle. The potential increase in protein biosynthesis combined with the downregulation of the TCA cycle, which also corresponds with the upregulation of FA biosynthesis, is crucial for cancer cells to maintain their proliferative demands as proteins and FA components are necessary during cell division [38]. Furthermore, TCA cycle downregulation may show that cancer cells, as they develop to more advanced stages, increasingly rely on glycolytic pathways over oxidative phosphorylation [3]. These alterations in amino acid levels and their respective pathways were previously found in other BC metabolomic studies in which different tumor grades were compared in vitro and in human biofluids, namely urine and blood [34,35,39,40,41,42,43,44,45,46,47].

Noticeably, methionine has not been previously reported as a differentially altered amino acid between BC grades. In this study, we observed that methionine metabolism was found to be one of the most important discriminatory pathways comparing HG and LG BC cells (Figure 3). This demonstrates that this pathway is altered towards the production of methionine, which is an essential amino acid that functions as an intermediate for protein synthesis, in transmethylation reactions as a methyl donor to S-adenosyl methionine (SAM) and in detoxifying processes, as well as an important compound for angiogenesis [48]. Moreover, the increase in methionine levels in HG cancer cells may also be associated with the downregulation of the TCA cycle. This is a novel finding that should be considered in future validation and translational studies with human samples.

## 4. Conclusions

In this study, we used an in vitro metabolomics approach to evaluate alterations in the endometabolome signatures of bladder cancer cells with distinct tumor grades. The bottom-up approach was selected due to its easily controllable setup and lower complexity compared to other disease models, therefore providing better insight into the metabolic changes between the two types of BC cells. The results obtained in this study regarding the endometabolome analysis of HG and LG cancer cells allow us to propose some metabolic alterations occurring during tumor progression in the bladder. Major changes were found in energy-related metabolic pathways, namely FA biosynthesis, amino acid metabolism, and ammonia recycling. Differences in FA levels between HG and LG cancer cell lines may reflect the distinct reliability in β-oxidation to generate energy. The same applies to amino acid metabolism along with ammonia recycling reflecting alterations in protein biosynthesis and the TCA cycle towards the support of tumor cell growth and proliferation. Furthermore, methionine stands out as an amino acid that, to our best knowledge, has never been reported before in in vitro studies comparing HG and LG bladder cancer, making this metabolite a novel finding to be validated. Overall, the results from our study may contribute to the discovery of promising biomarkers applicable to the categorization of the LG and HG forms of BC and the development of new therapeutic approaches, pending further investigation and validation.

## Figures and Tables

**Table 1:** List of metabolites selected as important for the discrimination between HG (J82) and LG (5637) bladder cancer cells. Values for ES, ES_SE_, *p*, and AUC are indicated for each metabolite.

Metabolite	HG J82 vs LG 5637			
	ES (±ES_SE_) *^a^*		*p*-Value *^b^*	AUC
**Amino acids and derivatives**				
Glycine	↓	−1.51 (±1.30)	1.59 × 10^−2^	0.960
Aspartic acid	↑	2.13 (±1.46)	1.59 × 10^−2^	0.960
Leucine	↑	1.48 (±1.29)	3.97 × 10^−2^	0.880
Methionine	↑	1.46 (±1.29)	3.17 × 10^−2^	0.920
Valine	↑	1.63 (±1.33)	3.17 × 10^−2^	0.920
**Fatty Acids**				
Myristic acid	↓	−4.50 (±2.27)	7.90 × 10^−3^	1.000
Palmitic acid	↓	−3.28 (±1.82)	7.90 × 10^−3^	1.000
Palmitoleic acid	↓	−4.46 (±2.25)	7.90 × 10^−3^	1.000

AUC, area under the curve; ES, effect size; ↑, metabolites increased; ↓, metabolites decreased in the endometabolome of HG vs. LG cancer cells. *^a^* ES determined as described in Reference [30]; *^b^* 95% significance level (*p* < 0.05).

## Supplementary Materials

The following are available online at http://www.mdpi.com/2218-1989/9/1/18/s1, Figure S1: Representative full scan of GC-MS chromatograms obtained from the endometabolome analysis of (A) 5637 and (B) J82 cell lines., Figure S2: PCA scores scatter plot obtained for the GC-MS chromatograms obtained from the endometabolome analysis of the cancer cell lines 5637 (*n* = 5, low-grade, TCC) and J82 (*n* = 5, high-grade, TCC). The ellipses indicate the 95% confidence limit of the model., Table S1: List of all metabolites identified in the endometabolomes of the cancer cell lines 5637 (LG) and J82 (HG). The identification of the metabolites is based on the NIST (2014) and standards. RT, characteristic ions (*m*/*z*), identification method with reverse % of match from NIST/standards when used, CAS registry number and HMDB code (when available) are indicated for each compound, as well as the main pathways in which they are involved and BC studies where they were previously found (when reported).
