# Supplementary material for: GC-MS Metabolomics Reveals Distinct Profiles of Low- and High-Grade Bladder Cancer Cultured Cells
Source: Metabolites. 2019 Jan 18;9(1):18. doi: 10.3390/metabo9010018 (PMC6359170; doi:10.3390/metabo9010018)
Supplement: Supplementary file 1 [file metabolites-09-00018-s001.pdf]

Supplementary Information

GC-MS metabolomics reveals distinct profiles of low- and high-grade bladder cancer cultured cells

Daniela Rodrigues[1,*], Joana Pinto[1], Ana Margarida Araújo[1], Carmen Jerónimo[2,3], Rui Henrique[2,3,4], Maria de Lourdes Bastos[1], Paula Guedes de Pinho[1], Márcia Carvalho[1,5,*]

[1]UCIBIO/REQUIMTE, Department of Biological Sciences, Laboratory of Toxicology, Faculty of Pharmacy, University of Porto, Porto, Portugal

[2]Cancer Biology & Epigenetics Group, Research Center (CI-IPOP) Portuguese Oncology Institute of Porto (IPO Porto), Porto, Portugal.

[3]Department of Pathology and Molecular Immunology-Biomedical Sciences Institute (ICBAS), University of Porto, Porto, Portugal.

[4]Department of Pathology, Portuguese Oncology Institute of Porto (IPO Porto), Porto, Portugal.

[5]UFP Energy, Environment and Health Research Unit (FP-ENAS), University Fernando Pessoa, Porto, Portugal

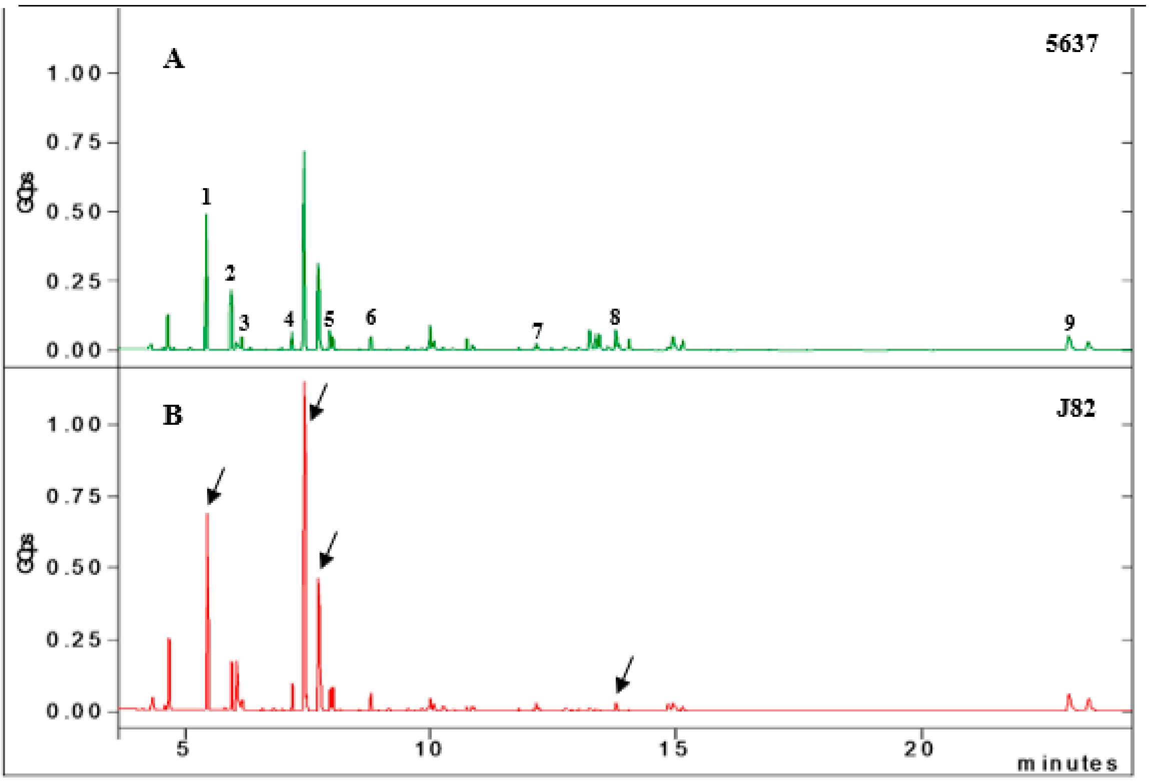

Supplementary Figure S1. Representative full scan of GC-MS chromatograms obtained from the endometabolome analysis of (A) 5637 and (B) J82 cell lines. Legend: 1 – Lactic acid (2TMS); 2 – L-alanine (2TMS); 3 – Glycine (2TMS); 4 – L-valine (2TMS); 5 – L-isoleucine (2TMS); 6 – L-threonine (3TMS); 7 – Myristic acid (TMS); 8 – Palmitic acid (TMS); 9 – Cholesterol (TMS). TMS, Trimethylsilyl (derivative). The arrows indicate the peaks whose differences are higher between the two BC cell lines.

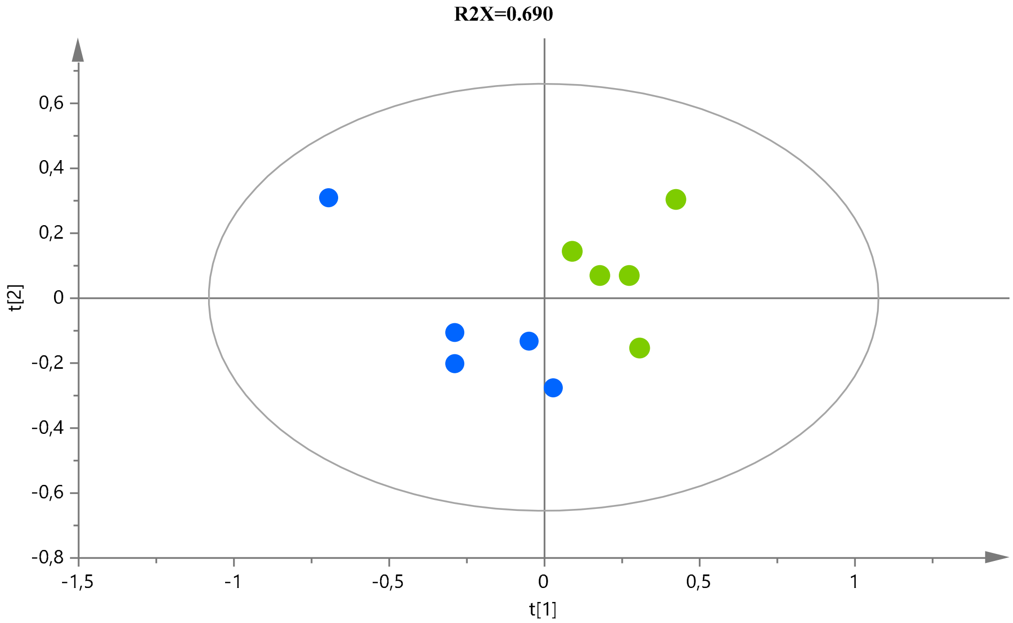

Supplementary Figure S2. PCA scores scatter plot obtained for the GC-MS chromatograms obtained from the endometabolome analysis of the cancer cell lines 5637 (n=5, low-grade, TCC, ●) and J82 (n=5, high-grade, TCC, ●). The ellipses indicate the 95% confidence limit of the model.

Supplementary Table S1. List of all metabolites identified in the endometabolomes of the cancer cell lines 5637 (LG) and J82 (HG). The identification of the metabolites is based on the NIST (2014) and standards. RT, characteristic ions (*m/z*), identification method with reverse % of match from NIST/standards when used, CAS registry number and HMDB code (when available) are indicated for each compound, as well as the main pathways in which they are involved and BC studies where they were previously found (when reported).

Metabolite	RT	Characteristic ions (m/z)	ID (R.Match/ Std)	CAS	HMDB code	Pathways	BC studies
Amino acids and derivatives							
**β-Alanine (2TMS)**	6.91	102	874	107-95-9	HMDB00056	Amino acid metabolism; Pyrimidine metabolism	NF
**Glycine (TMS/2TMS)**	4.12/6.17	75+132/75+102	945/932/Std	56-40-6	HMDB00123	Amino acid, Purine and Glutathione metabolisms; Bile acid biosynthesis	*In vitro:* ↑ (1); ↓ (2)<br>*Ex vivo* tissues: ↑ (3)<br>*Biofluids*: ↓ (4)
**α-Aminobutyric acid (2TMS)**	6.71	130	876	1492-24-6	HMDB00452	Amino acid metabolism	*Ex vivo* tissues: ↑ (5)
**L-5-Oxoproline (2TMS)**	10.00	156	940/Std	98-79-3	HMDB00267	Glutathione metabolism	NF
**L-Alanine (TMS/2TMS)**	4.11/5.95	75+103/116+117	905/959/Std	56-41-7	HMDB00161	Amino acid metabolism; Urea cycle	*In vitro:* ↑ (2, 6)<br>*Ex vivo* tissues: ↑ (3); ↓ (5)
**L-Aspartic acid (2TMS/3TMS)**	9.15/9.55	75+160<br>232	711/801/Std	56-84-8	HMDB00191	Amino acid and Purine metabolisms; Urea cycle	*In vitro:* ↑ (2)<br>*Ex vivo* tissues: ↑ (3)
**L-Glutamic acid (3TMS)**	10.75	128+246	918/Std	56-86-0	HMDB00148	Amino acid metabolism	*Ex vivo* tissues: ↑ (3)<br>*Biofluids*: ↑ (7)
**L-Isoleucine (2TMS)**	7.95	158+218	938/Std	73-32-5	HMDB00172	Amino acid metabolism	*In vitro:* ↑ (2)<br>*Ex vivo* tissues: ↑ (3, 5)<br>*Biofluids*: ↓ (4)
**L-Leucine (TMS)**	6.56	86	890/Std	61-90-5	HMDB00687	Amino acid metabolism	*Ex vivo* tissues: ↑ (3, 5)

							Biofluids: ↓ (4); ↑ (8)
**L-Methionine (TMS)**	9.10	56+104	832/Std	63-68-3	HMDB00696	Amino acid metabolism	NF
**L-Phenylalanine (TMS/2TMS)**	10.27/10.86	120+146 192+218	889/923/Std	63-91-2	HMDB00159	Amino acid metabolismo	*In vitro:* ↑ (2) *Ex vivo* tissues: ↑ (3, 5) *Biofluids*: ↓ (4, 9, 10); ↑ (8)
**L-Proline (TMS/2TMS)**	6.79/8.01	70+103 142+143	853/929/Std	147-85-3	HMDB00162	Amino acid metabolismo	*In vitro:* ↑ (2)
**L-Serine (2TMS/3TMS)**	7.60/8.56	116 204+218	873/933/Std	56-45-1	HMDB00187	Amino acid metabolismo	*Ex vivo* tissues: ↑ (5)
**L-Threonine (3TMS)**	8.79	117+218	957/Std	72-19-5	HMDB00167	Amino acid metabolismo	*In vitro:* ↑ (2)
**L-Valine (2TMS)**	7.17	144+218	931/Std	72-18-4	HMDB00883	Amino acid metabolismo	*In vitro:* ↑ (2) *Ex vivo* tissues: ↑ (3, 5) *Biofluids*: ↑ (7, 11)
Amino alcohol							
**Ethanolamine (2TMS)**	5.10	102	885	141-43-5	HMDB00149	Phospholipid biosynthesis	NF
Fatty Acids							
**10-Heptadecenoic acid (TMS)**	14.27	75+117	814	NA	HMDB60038	NA	NF
**11-Eicosenoic acid (TMS)**	16.81	75+117+129	814	2462-94-4	HMDB34296	NA	NF
**Arachidonic acid (TMS)**	16.31	75+79	846	506-32-1	HMDB01043	Arachidonic acid metabolism; Biosynthesis of unsaturated fatty acids	NF
**Heptadecanoic acid (TMS)**	14.42	116+327	871	506-12-7	HMDB02259	NA	*Biofluids*: ↑ (12)

**Myristic acid (TMS)**	12.48	75+117+129+132+285	912	544-63-8	HMDB00806	FA biosynthesis	NF
**Palmitic acid (TMS)**	13.78	75+117+129+313	949/Std	57-10-3	HMDB00220	FA and Glycerolipid metabolism; Bile acid biosynthesis	*Ex vivo* tissues: ↓ (5)
**Palmitoleic acid (TMS)**	13.65	75+117+129+311	918	373-49-9	HMDB03229	NA	NF
**Pentadecanoic acid (TMS)**	13.14	117+299	838	1002-84-2	HMDB00826	NA	NF
**Stearic acid (TMS)**	15.16	75+117	948/Std	57-11-4	HMDB00827	Biosynthesis of unsaturated fatty acids	NF
Monosaccharide							
**Dihydroxyacetone (2TMS)**	7.24	103+189	801	96-26-4	HMDB01882	Inositol phosphate metabolism	*Biofluids*: ↓ (12)
Organic acids and derivatives							
**Benzoic acid (TMS)**	7.53	105+137+179	809/Std	65-85-0	HMDB01870	NA	NF
**Glycolic acid (2TMS)**	5.61	75	851	79-14-1	HMDB00115	NA	NF
**Lactic acid (2TMS)**	5.45	117+191	949/Std	79-33-4	HMDB00190	Glycolysis/Gluconeogenesis; Pyruvate metabolism	*In vitro:* ↑ (2, 6) *Ex vivo* tissues: ↑ (3) *Biofluids*: ↑ (7, 12, 13), ↓ (4)
**Pyruvic acid (2TMS)**	5.77	148+217	892/Std	127-17-3	HMDB00243	Amino acid metabolism; Glycolysis/ Gluconeogenesis; Pentose Phosphate Pathway; TCA cycle	*In vitro:* ↓ (2, 6) *Biofluids*: ↑ (14)
**Succinic acid (2TMS)**	8.14	75	871/Std	110-15-6	HMDB00254	Amino acid metabolism; Pyruvate metabolism; TCA cycle	*Biofluids*: ↑ (13, 14)

Organic compounds							
**Uracil (2TMS)**	8.37	99+241	841	66-22-8	HMDB00300	Pyrimidine metabolism	*Ex vivo* tissues: ↑ (5)
**Urea (2TMS)**	7.53	189	918/Std	57-13-6	HMDB00294	Amino acids metabolism, Purine and Pyrimidine metabolisms	NF
Sterol							
**Cholesterol (TMS)**	23.01	75+129	925/Std	57-88-5	HMDB00067	Steroid biosynthesis; Bile acid biosynthesis	NF
Sugar alcohols							
**Erythritol (4TMS)**	9.84	217	812	149-32-6	HMDB02994	NA	*In vitro:* ↑ (2) *Biofluids*: ↑ (12)
**Myo-Inositol (6TMS)**	14.06	217+305	928/Std	87-89-8	HMDB00211	Inositol phosphate metabolism; Galactose metabolism	*In vitro:* ↓ (1); ↑ (2) *Ex vivo* tissues: ↑ (3)
**Scyllo-Inositol (6TMS)**	13.39	217	813/Std	488-59-5	HMDB06088	Inositol phosphate metabolism	NF
Unknown							
**Unknown 1**	9.75	144+188	-	-	-	-	-

CAS, Chemical Abstracts Service; CSF, Cerebrospinal Fluid; ER, Endoplasmic reticulum; FA, Fatty Acid; HMDB, Human Metabolome Database; ID, Identification; NA, not available; NF, not found; RI, Retention index; RT, Retention time; Std, Standard; TCA, tricarboxylic acid
